# Effects of dietary supplementation with multispecies probiotics on intestinal epithelial development and growth performance of neonatal calves challenged with *Escherichia coli* K99

**DOI:** 10.1002/jsfa.11791

**Published:** 2022-02-09

**Authors:** Yan‐yan Wu, Cun‐xi Nie, Chunsheng Xu, Rui‐qing Luo, Hong‐li Chen, Jun‐li Niu, Xue Bai, Wenju Zhang

**Affiliations:** ^1^ College of Animal Science and Technology Shihezi University Shihezi China; ^2^ Xinjiang Tianshan Junken Animal Husbandry Co. Ltd Shihezi China

**Keywords:** neonatal calves, diarrhea, probiotic, intestinal development

## Abstract

**BACKGROUND:**

Probiotics exhibit antibiotic properties and are capable of treating certain bacterial infections, including diarrhea. Therefore, the aim of this study is to investigate the effects of dietary supplementation with multispecies probiotic (MSP) on diarrhea, average daily gain (ADG) and intestinal development of neonatal calves challenged with *Escherichia coli* K99.

**RESULTS:**

Thirty‐six neonatal Holstein calves were randomly assigned to three treatment groups. After *E. coli* K99 challenge, calves in the control (C) and MSP treatment groups had significantly higher ADG and feed efficiency, and significantly lower fecal scores than those of calves in the diarrhea (D) group. The mean time of diarrhea resolution was 4.5 and 3.1 days for calves in the D and MSP treatment groups, respectively. Furthermore, the structures of the various segments (duodenum, jejunum and ileum) of the small intestine of the calves, activities of several small intestinal enzymes, and expression of several energy metabolism‐related genes in the small intestine segments were significantly affected by MSP treatments.

**CONCLUSION:**

Dietary supplementation of MSP had a positive effect in treating calf diarrhea; it improved ADG and feed efficiency and promoted development of the small intestine. © 2022 The Authors. *Journal of The Science of Food and Agriculture* published by John Wiley & Sons Ltd on behalf of Society of Chemical Industry.

## INTRODUCTION

Neonatal calf diarrhea is one of the most challenging clinical diseases preventing sustainable cattle production, and the detrimental effect on cattle herds can cause significant economic losses.^1^ Calf diarrhea is caused by infectious and non‐infectious factors,^2^ and infectious diarrhea is the predominant cause of morbidity and mortality in neonatal calves worldwide. Enterotoxigenic *Escherichia coli* (ETEC) is one of the major pathogens associated with neonatal calf diarrhea in the first week of life. The main factors affecting the pathogenicity of ETEC are fibrin antigens, mainly K99 (*E. coli* K99+) and heat‐stable enterotoxin.^3^ The fimbrial antigen promotes the adhesion of bacterial cells to the small intestine,^3^ whereas the heat‐stable enterotoxin causes intestinal mucosal damage in animals.^4^, ^5^

Bacterial enterotoxins cause diarrhea by increasing the osmolality of the intestinal lumen, which results in fluid accumulation in the intestines and diarrhea.^6^ The course of the disease is rapid, progressing from reduced absorption of essential nutrients, weight loss, weakness and diarrhea, to severe dehydration and death in less than 24 h.^7^ Moreover, calves experience acute symptoms of dehydration and metabolic acidosis, leading to chronic exhaustion and negative energy balance, which continues during the diarrhea period.^8^ To alleviate these problems, antibiotics have been widely used in animal production, which has led to drug resistance and drug residues in animal products, causing severe public health implications.^9^, ^10^ Owing to the emergence of antimicrobial‐resistant bacterial strains, the European Union^11^ and China^12^ prohibited the use of antimicrobial growth promoters in livestock nutrition. Therefore, the development of novel and multiple antidiarrheal agents including probiotics and the assessment of their effectiveness are now crucial in dairy farming.^13^

Probiotics are considered an ideal antibiotic substitute for treating bacterial infections.^14^, ^15^ Supplementing the diets of neonatal calves with probiotics can stimulate body growth^16^, ^17^ and organ development,^18^ reduce the incidence of diarrhea,^17^ and improve animal welfare and health,^19^ providing long‐lasting benefits for sustainable milk production and metabolic profiling.^20^ Studies in neonatal calves clearly reveal the importance of probiotic supplementation for improving the performance and health of Holstein calves^21^, ^22^ in terms of the immunity, expression of nutrient transporter genes, gut morphometry and microbiota of calves.^17^, ^23^, ^24^ However, the effects of probiotics on small intestine development in neonatal calves have not been investigated thoroughly.

Cell membrane integrity in the small intestinal mucosal epithelium is important for maintaining the structure and function of the small intestine, which depends on membrane protein functions of the constituent cells.^25^ Special proteins, including Na^+^‐K^+^‐ATPase and Ca^2+^‐Mg^2+^‐ATPase, are located within cell membranes and regulate ion transport to maintain ion balance inside and outside of cells.^26^ Abnormal functioning of ATPases with abnormal degrees of enzyme dysfunction is positively correlated with disease severity.^27^ Furthermore, the systemic and local somatotropic axis is involved in diet‐dependent regulation of intestinal development in pre‐weaned calves.^28^, ^29^, ^30^ Intestinal mucosa tissue contains insulin‐like growth factor (IGF) and insulin receptors that mediate IGF1 and IGF2 function, and insulin action in the intestine.^31^, ^32^ Moreover, the growth‐stimulating effects of probiotics seem to be regulated by the local IGF system.^33^ However, the effects of multispecies probiotics (MSP) supplementation on the regulation of the local IGF system, and gluconeogenic enzymes such as pyruvate carboxylase (PC) and cytosolic and mitochondrial phosphoenolpyruvate carboxykinase (PCK1 and PCK2) in the small intestines of calves, are yet to be investigated. In addition, the influence of MSP on gene expression in the context of energy metabolism in small intestinal mucosa is not well understood.

Local glucose production in the intestine may contribute to glucose homeostasis;^34^, ^35^ moreover, an increased expression of gluconeogenesis‐related genes in dairy cows^36^ suggested that local expression may also be affected by MSP. In the present study, we investigated the effects of MSP supplementation for 3 weeks on small intestinal development of neonatal calves challenged with *E. coli*. We hypothesize that MSP supplementation stimulates intestinal development and influences the IGF system and glucose metabolism‐related genes in the small intestinal mucosa in neonatal calves challenged with *E. coli*.

## MATERIALS AND METHODS

### Preparation of MSP complex

Three probiotic strains, namely *Lactobacillus acidophilus* S5,^37^ *Bacillus subtilis* (No. Bzg988118)^38^ and *Saccharomyces cerevisiae* (SHZ2017), along with the *Escherichia coli* K99 (O38), were provided by the Biological Feed Laboratory of the College of Animal Science and Technology, Shihezi University, China. The inhibitory effect of the three probiotics on *E. coli* K99 growth has been reported earlier via *in vitro* analyses.^39^

### Animal experiment, husbandry and diets

MSP was received from the source in the form of freeze‐dried powder and was mixed with fresh cow's milk for further use and experimentation. Thirty‐six neonatal Holstein bull calves with an average body weight of 40.1 ± 0.6 kg were randomly assigned to three treatment groups, according to weight and age (12 calves per treatment). The treatment groups included the control group (C), in which the calves were orally administered sterile saline (30 mL; not challenged with *E. coli* O78:K99); the diarrhea group (D), in which the calves were orally challenged with *E. coli* O78:K99 (30 mL; 1.0 × 10^9^ CFU mL^−1^)^13^ and were given antibiotic support therapy (intramuscular gentamicin 20 mL d^−1^ for 2 days); and the MSP group (MSP), wherein the calves were orally challenged with *E. coli* O78:K99 (30 mL; 1.0 × 10^9^ CFU mL^−1^) and were fed basal diet supplemented with MSP (7.0 × 10^9^ CFU g^−1^; 2 g d^−1^ daily. According to a previous research, the best effect was obtained in calves that were fed a diet containing 2 g d^−1^ MSP (1 g MSP comprises *L. acidophilus* 3 × 10^9^ CFU, *B. subtilis* 3 × 10^9^ CFU and *S. cerevisiae* 1 × 10^9^ CFU).^40^ Calves in the C and D groups were fed basal diets without MSP. The basal diet (Supporting Information Table S1) was free of antibiotics. The calves were fed the experimental diets for 21 days. The calves were orally challenged with *E. coli* O78:K99 when they were 1 day old (day 1), and antibiotic support therapy and probiotic supplementation was started on the following day.

The calves were bedded on straw and housed in individual pens (1.8 × 1.4 × 1.2 m) separated with iron fences to avoid cross‐contamination. The calves were fed 4 L colostrum (pasteurized at 60 °C for 1 h) using a bottle within 1 h of birth. Thereafter, they were weighed using an electronic scale, and colostrum intake was subtracted to determine the birth weight. The serum IgA concentration for passive transfer of immunity was 0.59 ± 0.2 mg mL^−1^. The calves were fed milk twice daily using two equal‐volume plastic buckets at 07:00 and 18:00. Once the calves were 5 days old, the volume of feed was increased to 6 L d^−1^ (3 L per meal) of milk produced in the same farm and pasteurized at 60 °C for 1 h. On day 6, the volume of feed was increased to 7 L d^−1^ (3.5 L per meal) and to 8 L d^−1^ (4 L per meal) from days 7 to 26. Starter concentrates were provided by Xinjiang Urumqi Zhengda Feed Co. Ltd (Urumqi, China) and were fed to the calves since day 4. All calves received the same colostrum and milk.

### Sample collection and analysis

Feed intake and fecal score of each calf was recorded daily. The body weight and average daily gain (ADG) of each calf was calculated by dividing the total weight gained by the calf during the study period by the number of days. The final weight of each calf at the end of the study period was recorded in the morning after the test ended. The dry matter intake (DMI) of milk and starter diet was also recorded throughout the study period. A standard health scoring system^41^ was used to obtain the fecal scores every morning at 10:00 am for 21 consecutive days. Fecal consistency was scored on the following scale: 0 = normal; 1 = half‐shaped and pasty; 2 = loose but staying on the mat; and 3 = watery, sieving through the mat. A case of diarrhea was noted when the fecal score was 2 or higher.^42^ Antibiotic supportive therapy and MSP treatment were performed on the calves, as described earlier.

On day 26, 18 Holstein calves (six in each group) were anesthetized using 4 g kg^−1^ sodium pentobarbital solution as per their body weight, and euthanized by jugular vein bloodletting. The intestinal mucosal tissue and digesta samples from the duodenum, jejunum (middle), ileum (30 cm from the ileum–cecum junction), cecum (middle), colon (middle), and rectum (proximal) were collected in 2 mL sterile enzyme‐free cryotubes, which were immediately immersed in liquid nitrogen for rapid freezing and stored at −80 °C until further analysis. The intestinal digesta samples were used for enzyme activity analysis, whereas mucosal samples were used for gene expression analysis.

Additionally, samples of the proximal part of the duodenum (5 cm), middle part of the jejunum (2–3 cm) and middle part of the ileum (2–3 cm) were collected, rinsed using normal saline, followed by dehydration and fixing in 10 g kg^−1^ formaldehyde for subsequent morphology analysis. The collection process was performed under aseptic conditions, and all samples were collected within 20 min after euthanasia.^43^

Subsequently, the fixed intestinal samples were stained with hematoxylin–eosin (HE), according to the method described by Schäff *et al*.,^44^ for histological observation. Briefly, samples of each intestinal segment were cut into ten sections, stained with HE and observed using an ML‐50 microscope (Motic Deutschland GmbH, Wetzlar, Germany). The images were visualized at a magnification of 40× to observe and measure the villus height, villus width, crypt depth and membrane thickness of different intestinal segments. The villus height/crypt depth ratio (V/C) was determined.

The enzyme activities of Ca^2+^‐Mg^2+^‐ATPase, Na^+^‐K^+^‐ATPase, bovine creatine kinase (CK), bovine maltase (maltase), bovine lactase (lactase), bovine lipase (lipase), bovine trypsin (Try) and bovine amylase (AMS) in the intestinal mucosa of the calves were determined using enzyme‐linked immunosorbent assay detection kits (Shanghai Jianglai Bio, China).

### Real‐time polymerase chain reaction (PCR) analysis of intestine mucosa

Tissue preparation for mRNA quantification was performed as described by Schäff *et al*.^44^ Total RNA was extracted from homogenized powdered mucosa tissue using Trizol reagent (Life Technologies, Darmstadt, Germany). The integrity and quality of the total RNA extract was verified using gel electrophoresis, and the RNA integrity number (RIN) was determined using Agilent RNA 6000 Nano Kit (Bioanalyzer 2100, Agilent, Hamburg, Germany). The mean RIN for the duodenum, jejunum, and ileum were (mean ± SE) 7.5 ± 0.2, 8.6 ± 0.1 and 8.7 ± 0.2, respectively. The quantity and quality of total RNA were also determined by measuring the optical density at 260 and 280 nm using a spectrophotometer (NanoPhotometer, Implen GmbH, Munich, Germany). For cDNA synthesis, 750 ng RNA was reverse‐transcribed with 200 U M‐MLV‐Reverse Transcriptase RNase (H−) Point Mutant (M‐MLV RT [H−], Promega, Madison, WI, USA) and 250 pmol random hexamer primer (Metabion International AG, Germany).

The synthesized cDNA was diluted at a ratio of 1:4 with diethyl pyrocarbonate water, and aliquots were stored at −80 °C. Specific primers (Supporting Information Table S2) were used to quantify the relative mRNA expression of growth hormone receptor (*GHR*), insulin‐like growth factor 1 (*IGF1*), insulin‐like growth factor binding protein 2 (*IGFBP2*), insulin‐like growth factor binding protein 3 (*IGFBP*3), insulin‐like growth factor 1 receptor (*IGF1R*), insulin receptor (*INSR*), *PC*, *PCK1*, *PCK2*, propionyl‐CoA carboxylase (*PCCA*), *SLC5A1*, lactase (*LCT*), maltase glucoamylase (*MGAM*), sucrase–isomaltase (*SI*), lactate dehydrogenase A (*LDHA*) and lactate dehydrogenase B (*LDHB*). Primers were designed with Primer3 version 0.4.0 or obtained from the literature as shown in Table S2. Relative expression of the target genes was determined using the 2^‐△△Ct^ method.^45^

### Statistical analyses

Data obtained from the study were analyzed using SAS software version 9.4 (SAS Institute Inc., Cary, NC, USA). Data regarding the effects of key factors and their interactions with parameters including ADG, DMI, feed efficiency (FE), initial weight, final weight at the end of the study period, total time until diarrhea resolution, mean diarrhea resolution time and enzyme activities were analyzed using the MIXED procedure of the SAS/STAT software. The intestinal permeability index data was analyzed using Microsoft Excel 2003 and SAS 8.2. Mean values were compared using the Student–Newman–Keuls test. Pearson's correlation analysis was performed to determine the relationships between calf mucosal development, small intestinal enzyme activities and mRNA expression of *GHR*. The SLICE statement of the MIXED procedure was used to conduct partitioned analyses of the least squares means (LSM) for the interactions. Mean values were considered significantly different at *P* < 0.05.

## RESULTS

### Effect of MSP treatment on growth performance and resolution of diarrhea

No deaths were recorded during the study. The ADG, FE and final weight of the animals were significantly affected by the treatments. Overall, calves in the C and MSP treatment groups showed significantly higher ADG, FE and final weights than calves in the D group. However, the DMI of the calves was not significantly affected by the treatments. The initial weight of the calves was not significantly different. The treatments (supportive antibiotic therapy) were administered to ten (83.3%) and two (16.2%) calves in the D and MSP groups, respectively. Additionally, the time until resolution of diarrhea was significantly affected by the treatments, with calves in the D group having the longest resolution time (4.5 days), followed by those in the MSP treatment group (3.1 days). Therefore, the calves in the MSP group showed faster resolution of diarrhea than calves in the D group (Table 1).

**Table 1:** Effects of multispecies probiotics (MSP) supplementation on growth performance of neonatal calves challenged by *E. coli* K99

Item	Treatment			SEM	*P*‐value^a^
	C	D	MSP		
Average daily gain (kg d^−1^)	0.78a	0.51b	0.67a	0.11	0.01
Dry matter intake (kg d^−1^)	1.16	1.08	1.15	0.09	0.52
Feed efficiency^b^ (%)	58.62a	47.22b	53.04a	6.15	0.01
Initial weight (kg)	41.2	42.2	41.6	0.19	0.34
Final weight (kg)	63.04a	56.88b	60.36a	4.21	0.04
Treatment^c^ after enrollment (%)	0	83.3 (10)	16.2 (2)	0.98	0.02
Time until resolution of diarrhea (days)	0	4.8	4	0.02	0.04
Mean resolution of diarrhea (days)	0	4.5	3.1	0.13	<0.01

^a^ Values are represented as least squares mean (LSM) with respective standard errors (SE); *n* = 6 per group.

^b^ Feed efficiency = [Average daily gain (kg d^−1^)/Dry matter intake (kg d^−1^)] × 100%. Values in the same row with different letters (a,b) differ significantly (*P* < 0.05).

^c^ Supportive or antibiotic therapy.

### Effect of MSP treatment on intestinal tract development and morphology

**Figure 1:**
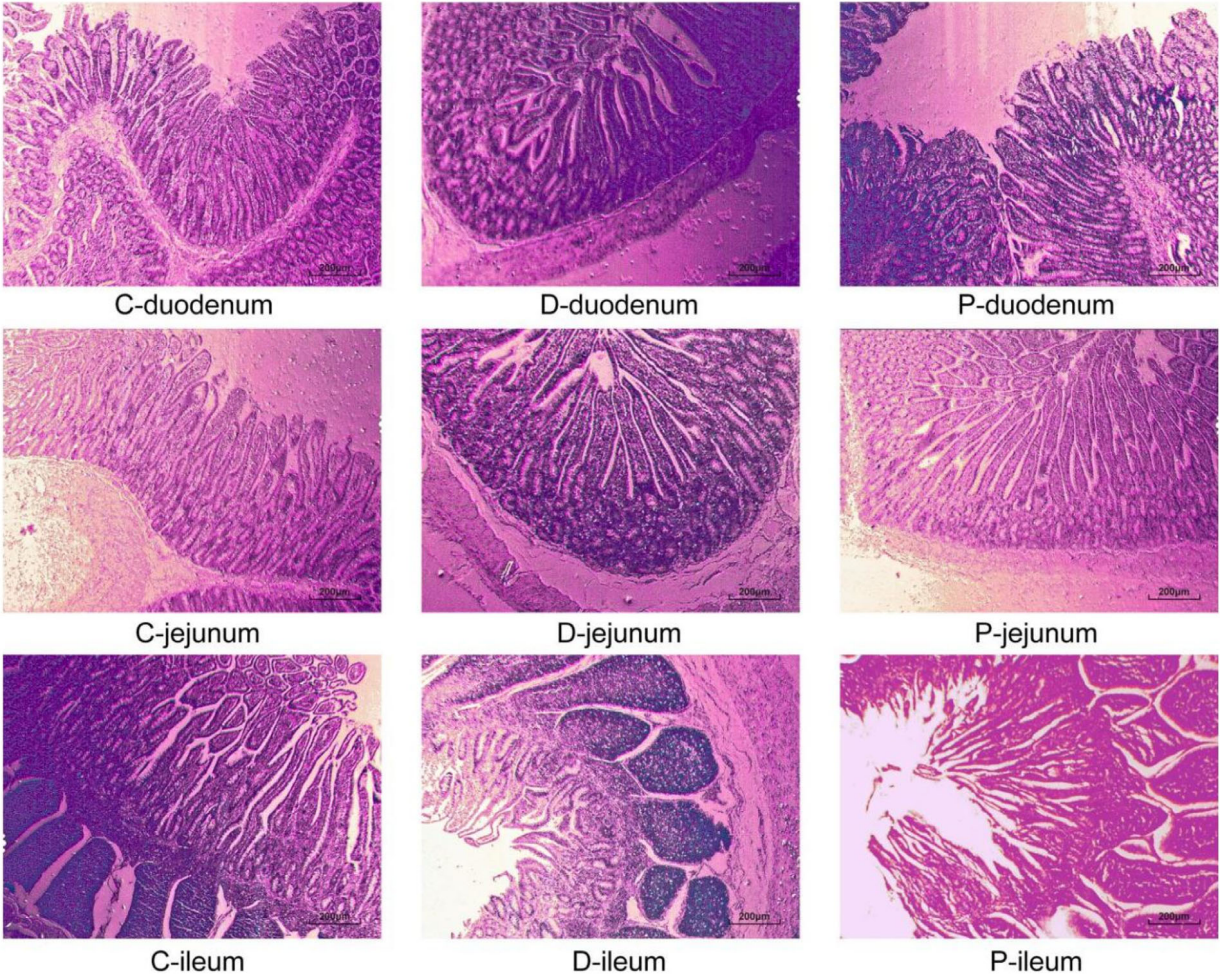
Effects of multispecies probiotics (MSP) supplementation on intestinal tissue morphology of neonatal calves (C, D and P represent the control, diarrhea and MSP groups, respectively).

The intestinal tract of the calves was significantly affected by the treatments. Calves in the MSP treatment group had significantly higher (*P* < 0.05) duodenal and jejunal villus height, duodenal crypt depth, and duodenal, jejunal and ileal intestinal mucosal thickness than those of the calves in the D group (Table 2). However, calves in the C treatment group had significantly higher ileal villus height and crypt depth, and duodenal and jejunal V/C ratio than the D and MSP treatment group calves (Table 2; Fig. 1).

**Table 2:** Effects of MSP supplementation on villi height, crypt depth and mucosal thickness of the duodenum, jejunum and ileum in neonatal calves

Item^a^	Treatment (Trt)^b^				*P*‐value	
	C	D	MSP	SEM	Gut segments	Trt
Villus height (μm)						
Duodenum	293.27ab	234.19b	346.18a	11.04	<0.01	<0.01
Jejunum	316.01b	291.27b	409.93a	14.74		<0.01
Ileum	406.29a	213.13b	265.08b	14.52		0.03
Crypt depth (μm)						
Duodenum	45.46b	151.59a	145.06a	4.96	<0.01	<0.01
Jejunum	118.8c	197.98a	155.71b	4.73		0.01
Ileum	170.48a	128.49b	130.55b	3.87		0.02
Villus height/crypt depth						
Duodenum	5.52a	1.97b	2.43b	0.23	<0.01	<0.01
Jejunum	2.55a	2.17ab	1.98b	0.09		0.02
Ileum	2.85	1.65	2.12	0.13		0.98
Intestinal wall thickness						
Duodenum	262.05b	242.93b	512.94a	14.99	<0.01	<0.01
Jejunum	437.07b	215.54c	551.94a	21.37		<0.01
Ileum	407.93b	216.84c	546.26a	11.37		<0.01

Different letters (a–c) within a row indicate significant differences (*P* ≤ 0.05).

^a^ Values are represented as least squares mean (LSM) with respective standard errors (SE); *n* = 6 per group.

^b^ Treatments: C, The control group; D, The diarrhea group.

### Effect of MSP treatment on enzyme activity in the small intestine

The treatments significantly affected the activities of the enzymes in the different segments of the small intestine (Table 3). Overall, calves in the C group had significantly higher (*P* < 0.01) intestinal Try and AMS activities, followed by calves in the MSP treatment and D groups. The duodenal, jejunal and ileal Ca^2+^‐Mg^2+^‐ATPase, Na^+^‐K^+^‐ATPase, CK, maltase, lactase, lipase, Try and AMS activities of calves were significantly affected by the treatments. However, calves in the C group showed the highest activities of almost all the enzymes.

**Table 3:** Effects of MSP supplementation on digestive enzyme and absorption enzyme activity of intestinal contents in neonatal calves

Item^a^ ^,^ ^b^	Treatment				*P*‐value	
	C	D	MSP	SEM	Gut segments	Trt
Ca^2+^‐Mg^2+^‐ATPase (U g^−1^)						
Duodenum	1.54a	0.87c	1.13b	0.07	0.46	<0.01
Jejunum	1.19a	0.82b	1.24a	0.06		0.01
Ileum	1.29a	0.87b	1.17a	0.06		0.02
Na^+^‐K^+^‐ATPase (U g^−1^)						
Duodenum	0.73a	0.49c	0.6b	0.02	0.39	<0.01
Jejunum	0.66a	0.49b	0.63a	0.01		0.02
Ileum	0.65a	0.48b	0.68a	0.01		0.04
CK (U g^−1^)						
Duodenum	1.02a	0.54b	0.92a	0.05	0.56	<0.01
Jejunum	0.92a	0.71b	1a	0.03		0.02
Ileum	1.07a	0.48c	0.84b	0.01		0.01
Maltase (U g^−1^)						
Duodenum	1.71a	0.73b	1.56a	0.04	0.66	<0.01
Jejunum	1.79a	0.8c	1.31b	0.1		0.02
Ileum	1.32ab	0.91b	1.42a	0.19		0.03
Lactase (U g^−1^)						
Duodenum	0.68a	0.51b	0.69a	0.03	0.88	0.01
Jejunum	0.79a	0.48c	0.59b	0.03		0.01
Ileum	0.76a	0.47c	0.58b	0.03		0.01
Lipase (U g^−1^)						
Duodenum	7.66a	3.93c	6.22b	0.38	0.06	<0.01
Jejunum	7.68a	5.16b	4.86b	0.21		<0.01
Ileum	6.85a	5.39b	6.26ab	0.2		0.03
Try (U g^−1^)						
Duodenum	1.74a	0.89c	1.6b	0.09	0.37	<0.01
Jejunum	1.57a	0.92c	1.23b	0.07		<0.01
Ileum	1.66a	0.7b	1.41a	0.1		0.02
AMS (IU g^−1^)						
Duodenum	2368.48a	1089.38b	2228.89a	144.89	0.11	<0.01
Jejunum	2642.06a	602c	1900.18b	218.9		<0.01
Ileum	2470.57a	1861.76b	2233.64a	74.28		0.02

Different letters (a,b) within a row indicate significant differences (*P* ≤ 0.05).

^a^ Values are represented as least squares mean (LSM) with respective standard errors (SE); *n* = 6 per group.

^b^ CK, creatine kinase; Try, trypsin; AMS, amylase.

### Effect of MSP treatment on gene expression in small intestine

**Figure 2:**
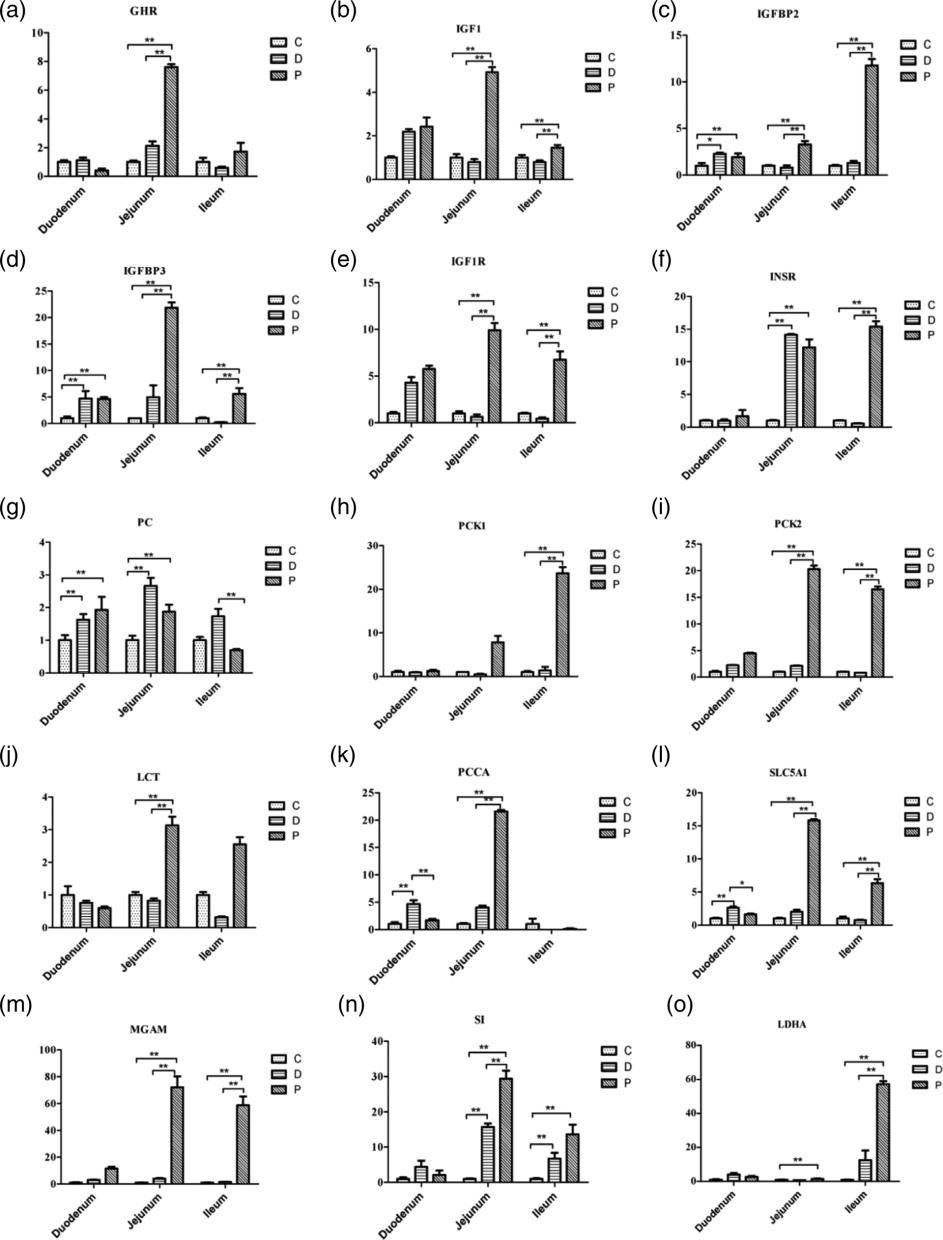
Effects of multispecies probiotics (MSP) supplementation on the expression of small intestinal enzyme‐encoding genes: (a) growth hormone receptor, *GHR*; (b) insulin‐like growth factor 1, *IGF1*; (c) insulin‐like growth factor binding protein 2, *IGFBP2*; (d) insulin‐like growth factor binding protein 3, *IGFBP3*; (e) insulin‐like growth factor 1 receptor, *IGF1R*; (f) insulin receptor, *INSR*; (g) pyruvate carboxylase, *PC*; (h) cytosolic phosphoenolpyruvate carboxykinase, *PCK1*; (i) mitochondrial phosphoenolpyruvate carboxykinase, *PCK2*; (j) lactase, *LCT*; (k) propionyl‐CoA carboxylase, *PCCA*; (l) sodium‐dependent glucose cotransporter 1, *SLC5A1*; (m) maltase glucoamylase, *MGAM*; (n) sucrase‐isomaltase, *SI*; (o) lactate dehydrogenase A, *LDHA*. *P < 0.05; **P < 0.01.

The treatments significantly affected the expression profiles of genes encoding the enzymes of the small intestine (Fig. 2). A significant decrease (*P* < 0.01) in the expression of genes related to the somatotropic axis and insulin was noted in the small intestines of calves in the D group, except *GHR* and *IGFBP3*. Duodenal expression of *GHR* genes was significantly lower (*P* < 0.01) in calves in the MSP treatment group than that in calves in the C and D groups. Duodenal mucosal expression of *MGAM* gene was significantly higher (*P* < 0.01) in calves in the MSP treatment group than that in calves in the C and D groups. The jejunal and ileal mucosal expression of *IGF1*, *IGFBP2*, *IGFBP3*, *IGF1R*, *PCK1*, *PCK2*, *SLC5A1*, *LCT*, *MGAM* and *SI* genes was significantly higher (*P* < 0.01) in calves in the MSP treatment group than that in calves in the C and D groups (Fig. 2, Supporting Information Table S3).

**Figure 3:**
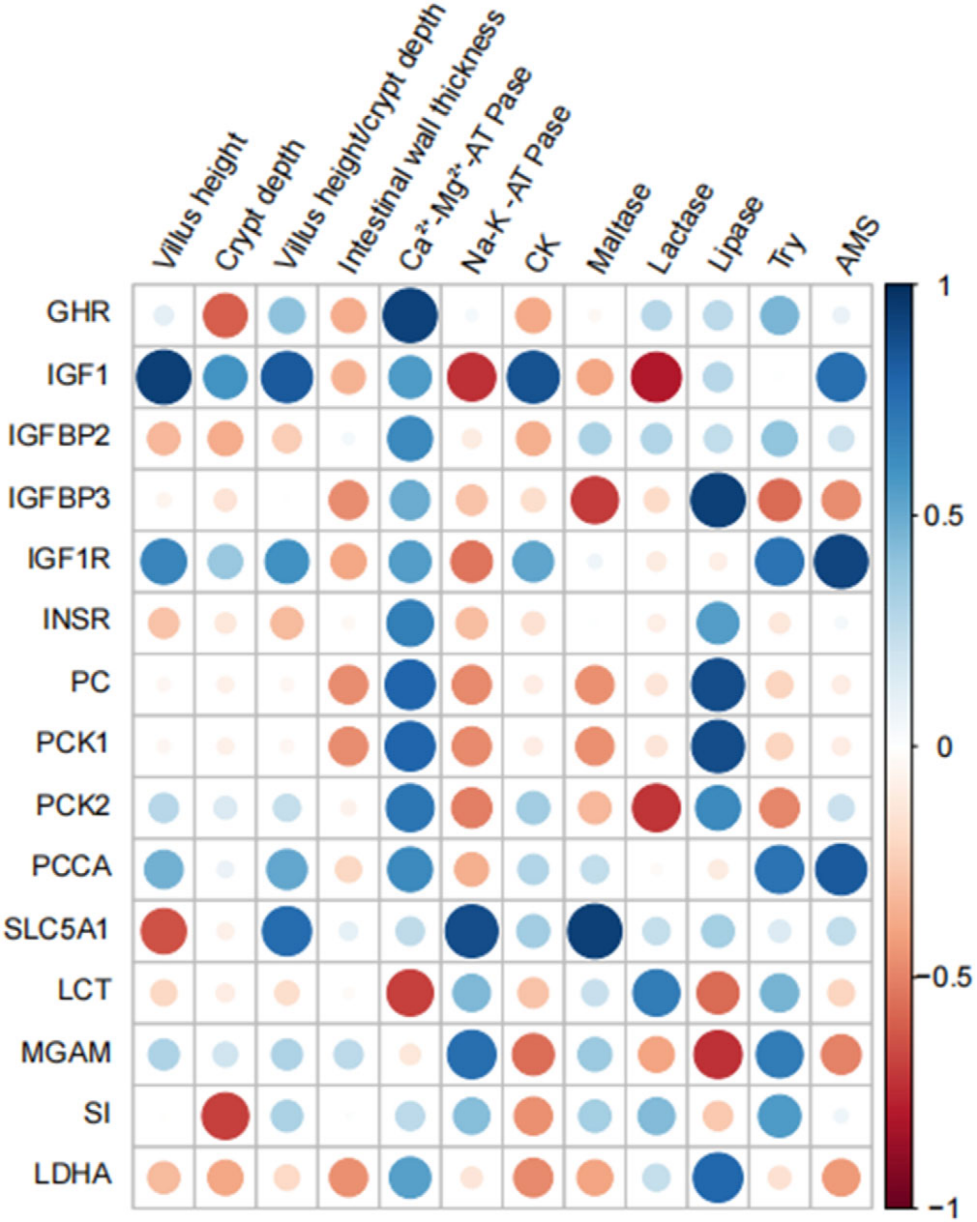
Pearson's correlation coefficients between the parameters describing the development of the intestinal tract and the expression levels of the enzyme‐coding genes of jejunum mucosa.

The results of the Pearson's correlation analysis showed that the expression of *GHR* gene was significantly and positively correlated (*r* = 0.92; *P* < 0.05) with the jejunal mucosal Ca^2+^‐Mg^2+^‐ATPase level. The expression of *IGF1* gene was significantly and positively correlated with jejunal mucosal villus height, V/C ratio and CK activity (*r* = 0.93, 0.93 and 0.86, respectively; *P* < 0.05). In addition, the expression of *SLC5A1* gene was significantly and positively correlated (*r* = 0.88; *P* < 0.01) with jejunal mucosal Na^+^‐K^+^‐ATPase activity. The expression levels of *IGFBP3*, *PC* and *PCK1* genes were significantly and positively correlated with jejunal mucosal lipase activity (*r* = 0.93, 0.88 and 0.88, respectively; *P* < 0.01), while the expression of *IGF1R* and *PCCA* genes was significantly and positively correlated with jejunal mucosal AMS activity (*r* = 0.91 and 0.83, respectively; *P* < 0.05). The expression of *PCK1* gene was significantly and negatively correlated (*r* = −0.86; *P* < 0.01) with duodenal mucosal CK activity. The expression of *IGFBP3* gene was significantly and negatively correlated (*r* = −0.95; *P* < 0.01) with duodenal intestinal wall thickness (Fig. 3; Supporting Information Table S4).

## DISCUSSION

Neonatal calf diarrhea is a major cause of calf death within a few days after birth, resulting in significant economic losses to the dairy industry. In the present study, we assessed the impact of MSP supplementation on a range of parameters associated with the health and growth of neonatal Holstein calves.

Our results showed that MSP supplementation improved the ADG and FE of the calves and reduced the duration of diarrhea, which may be attributed to the regulatory effects of the probiotics.^46^ The use of probiotics has shown some efficacy in reducing the duration of diarrhea in infants with acute infectious diarrhea,^47^ while the use of MSP as feed additive for animals has also shown promising results.^17^, ^48^ In calves, prevention of the incidence of calf diarrhea and improving ADG and FE has been the primary focus of research on probiotic species.^49^ In this context, previous studies have shown that probiotics can alleviate and shorten the duration of diarrhea and increase ADG after diarrhea.^17^, ^48^, ^50^ Furthermore, *L. acidophilus*,^51^ *B. subtilis* ^52^ and *S. cerevisiae* ^53^, ^54^ can independently improve calf growth performance by improving immune function and balancing the structure of intestinal microbiota.^55^, ^56^ However, MSPs are superior to single‐strain probiotics, especially for treating antibiotic‐associated diarrhea, enhancing resistance against bacterial infections and weight gain post enteritis, both in humans and animals.^17^, ^57^, ^58^ Our findings are consistent with these observations.

Successful management of dairy farms is vastly dependent on rearing healthy calves, and the optimal development of the intestinal tract is crucial for achieving this goal.^59^ Optimal functioning of the host intestinal barrier is a major contributor to intestinal health.^60^ It was thus necessary to analyze the structure and function of the intestinal barrier for effective prevention and treatment of diarrhea in calves. Diarrhea has been reported to impair intestinal barrier integrity and promote inflammatory immune response against symbionts.^13^, ^61^ Moreover, *E. coli* infection destroys the gut integrity of calves and seriously damages the intestinal function of animals, leading to a decline in their growth performance.^62^, ^63^, ^64^ It is also known that increase in villus height and V/C ratio are directly associated with increased epithelial cell turnover.^65^ Consistent with this expectation, the findings of the present study clearly reveal a significant increase in the jejunal villi heights of calves in the MSP treatment group. In addition, the growth‐stimulating effects of MSP and its impact on intestinal mucosal repair have been well described.^48^ Furthermore, dietary *L. acidophilus* positively influences gut morphology in animals, as demonstrated via morphometric evaluations. *Lactobacillus acidophilus* supplementation increased mucosal layer height by improving villus height, whereas crypt depth remained unaffected.^66^, ^67^ Also, *B. subtilis* supplementation can increase the ileal villus height and V/C ratio and serve as an antibiotic substitute for the intestinal ecosystem of weaned piglets.^68^ Dietary *B. subtilis* supplementation also upregulates the genes involved in the metabolic pathways related to intestinal microbiota maturation after intramuscular inoculation with *E. coli*.^69^ Furthermore, *S. cerevisiae* fermentation products (SCFP) improve the morphology of the gastrointestinal tract of dairy calves,^70^ likely resulting from reduction of jejunal crypt depth in the small intestine, along with reduction in the intestinal pH value, as observed in animals.^59^ In the present study, MSP supplementation also significantly increased the duodenal, jejunal and ileal villus heights and crypt depths in neonatal calves infected by *E. coli* K99, indicating that MSP‐supplemented diets can improve the development of the intestinal mucosa.

Moreover, it is possible that MSP supplementation can promote the secretion of Na^+^‐K^+^‐ATPase and Ca^2+^‐Mg^2+^‐ATPase in the small intestine, as evidenced by the higher intestinal activities of Na^+^‐K^+^‐ATPase and Ca^2+^‐Mg^2+^‐ATPase in calves in the MSP treatment group as compared to those in other treatment groups. Na^+^‐K^+^‐ATPase and Ca^2+^‐Mg^2+^‐ATPase are ubiquitous ion pumps that employ active transport of ions to establish the required gradients. To maintain normal gastrointestinal tissue function and prevent tissue damage, Na^+^‐K^+^‐ATPase and Ca^2+^‐Mg^2+^‐ATPase are key for active transport‐based maintenance of the host intestinal barrier. Reduced activities of such ATPases lead to dysfunction of the membrane ion pump and Na^+^, K^+^, Ca^2+^ and Mg^2+^ imbalance, eventually resulting in swelling and necrosis of gastric mucosal cells.^71^, ^72^ In most animal cells, Na^+^‐K^+^‐ATPases are cytoplasmic membrane glycoproteins that serve as carrier proteins involved in the active transport of Na^+^ and K^+^.^73^ Similarly, Ca^2+^‐Mg^2+^‐ATPases are a group of important enzymatic cytoplasmic membrane glycoproteins that function as ion transporters. A decrease in Ca^2+^‐Mg^2+^‐ATPase activity can reduce ion transport across the membrane barrier, causing Ca^2+^ to accumulate in the cytoplasm, resulting in abnormal cell morphology, structure and function.

The growth‐stimulating effect of probiotics treatment on the intestinal mucosa has been well described.^48^, ^59^, ^67^ The local IGF system may have a regulatory effect of on intestinal mucosa growth.^28^, ^32^ Particularly, the jejunal and ileal expression levels of *GHR*, *IGF1*, *IGFBP2*, *IGFBP3*, *IGF1R* and *INSR* genes have been reported to be higher than those of the duodenum. Our findings revealed that the expression of the *IGF* gene was positively correlated with the V/C ratio and negatively correlated with crypt depth, indicating that the local *IGF* system in the small intestinal mucosa had a positive relationship with villus development and an antagonistic relationship with crypt depth.^29^, ^30^, ^32^ Furthermore, the expression levels of the genes examined in the presented study (*GHR*, *IGF1*, *IGFBP2*, *IGFBP3*, *IGF1R*, *PC*, *PCK1*, *PCK2*, *LCT*, *PCCA*, *SLC5A1*, *MGAM* and *LDHA*) significantly affected the development of the intestinal epithelial cells of the calves. Our results also suggest that the use of antibiotics in treating calf diarrhea may cause some side effects, such as intestinal barrier dysfunction.^15^ In contrast, the dietary supplementation with probiotics was more effective in treating calf diarrhea, as observed earlier.^9^, ^74^ This observation was further supported by the positive association of lactase activity with the expression of *IGF* (i.e., *GHR*, *IGFBP2* and *INSR*) genes. Moreover, our results clearly revealed that insulin and IGF1 are not the only drivers of small intestine mucosal development, since villus size was also enhanced by dietary MSP supplementation. This effect of MSP was mediated by regulating the activities of systemic insulin and IGF. Bühler *et al*.^75^ reported that growth hormone treatment reduced the C/V ratio and increased the crypt depth in neonatal calves, whereas oral or systemic administration of IGF1 had no effect on the growth of the intestinal mucosa.

In the present study, the expression of most genes related to intestinal glucose metabolism was significantly different between different intestinal segments of neonatal calves challenged with *E. coli* K99, and were affected by dietary MSP supplementation. Additionally, glucose absorption was mainly mediated by sodium/glucose cotransporter (SLC5A1), which transports glucose to the epithelium.^76^ Moran *et al*.^77^ reported that *SLC5A1* expression increased along the crypt–villus axis of the small intestine. Consistent with this, we observed that *SLC5A1* gene expression showed a positive correlation with small intestinal villus height in calves in the MSP treatment group. This may be due to the higher number of SLC5A1 transporters in long villi, which may have enhanced the absorption of dietary sugars. The sugar‐absorbing activity of the jejunum is crucial for the functional recovery processes of the cattle suffering from diarrhea and that have damaged intestinal mucosa.^27^ Moreover, *SLC5A1* is a major Na^+^‐dependent glutamate (Glu) transporter expressed in the epithelial cells of pigs, and is characterized as the primary transporter of luminal l‐Glu across the enterocyte apical membrane.^77^ We observed that jejunal and ileal *SLC5A1* mRNA expression was positively correlated with villus height, and thus speculated that a higher *SLC5A1* expression in the epithelial cells of the small intestine promoted glutamate absorption, which was beneficial for calf growth and intestinal development.^76^

Electrolyte disturbances, such as acidemia and hyperkalemia are common in neonatal calves with diarrhea. Such imbalances negatively affect the cell's response to insulin, and may lead to disturbances in glucose, potassium and phosphorus homeostasis.^78^ The complications of neonatal calf diarrhea include dehydration, azotemia and the development of hyponatremic strong ion (metabolic) acidosis and varying degrees of hyperlactemia.^79^, ^80^ Hyperkalemia, commonly associated with calf diarrhea, can lead to skeletal muscle weakness, life‐threatening cardiac conduction abnormalities and arrhythmia.^81^, ^82^ As potassium is mainly an intracellular cation, hyperkalemia in calves with neonatal diarrhea is conventionally attributed to internal potassium imbalance caused by acidemia and is thus dependent on the degree of dehydration.^83^ Therefore, the intracellular buffering effect of hydrogen ions and impaired Na^+^‐K^+^‐ATPase activities are most likely the potential mechanisms mediating hyperkalemia during diarrhea. Since the renal perfusion is also reduced, the glomerular filtration rate seems to play a decisive role in the development of hyperkalemia. Nevertheless, other factors may still play important pathophysiological roles in the development of hyperkalemia in calves with diarrhea. In this study, MSP supplementation in the diet of neonatal calves infected with *E. coli* K99 significantly increased the jejunal mucosal Ca^2+^‐Mg^2+^‐ATPase and Na^+^‐K^+^‐ATPase activities, with calves in the D group having the lowest activities. This may be attributed to the regulatory effect of MSP on epithelial growth and development of the intestinal tract of neonatal calves challenged with *E. coli* K99.

We further noted that the *PCK2* gene expression in the proximal part of the small intestine was significantly lower in calves in the D treatment group than in calves in the MSP treatment group. We were unable to ascertain whether significant intestinal gluconeogenesis was present in the calves, similar to that in other species.^34^ However, intestinal gluconeogenesis can also be affected by nutrients and intestinal microbiome.^37^ Variation in feed intake of calves and intake of a more concentrated feed in the MSP treatment group may have influenced intestinal gluconeogenesis by providing volatile fatty acids (VFA), such as propionate, for glucose synthesis.^37^ Furthermore, a lower *LDHA* gene expression in the distal parts of the small intestine after MSP supplementation probably indicates lower lactate production from pyruvate in the enterocytes.

## CONCLUSIONS

This study demonstrates that MSP supplementation can improve the ADG of neonatal calves challenged with *E. coli* K99, improve intestinal epithelial cell growth (especially in the jejunum), and regulate intestinal energy metabolism to improve nutrient digestion and absorption. The local IGF system is involved in the regulation of intestinal growth and maturation, but the interaction of the systemic and local somatotropic axes needs further investigation. The findings of the study showed that MSP was more effective than antibiotics in repairing the intestinal structure of calves and in treating neonatal calf diarrhea caused by *E. coli*. Based on our analysis, we recommend MSP to be supplemented at a rate of 2 g d^−1^ to the diet of neonatal calves challenged with *E. coli* K99. Our findings provide a basis for the rational use of MSP supplements in calf production and may help reduce the use of antibacterial agents. However, further research is necessary to explore the functional modifications resulting from the changes in intestinal microbial homeostasis after MSP supplementation.

## CONFLICT OF INTEREST

The authors declare that the research was performed without any commercial or financial relationships that could be construed as potential conflicts of interest.

## Supporting information


**Supplemental Table S1.** Ingredient composition and nutrient levels of starter (DM basis)
**Supplemental Table S2**. Primer sequences and annealing temperature
**Supplemental Table S3.** The effect of MSP supplementation on the growth of calf mucosa and the relative expression of small intestinal enzyme activity mRNA
**Supplemental Table S4**. Pearson correlation coefficients between the growth of calf mucosa and the relative expression of jejunum enzyme activity mRNA
