# Supplementary material for: Effects of dietary supplementation with multispecies probiotics on intestinal epithelial development and growth performance of neonatal calves challenged with Escherichia coli K99
Source: J Sci Food Agric. 2022 Feb 9;102(10):4373–83. doi: 10.1002/jsfa.11791 (PMC9303730; doi:10.1002/jsfa.11791)
Supplement: Supplementary file 1 — Supplemental Table S1. Ingredient composition and nutrient levels of starter (DM basis) Supplemental Table S2. Primer sequences and annealing temperature Supplemental Table S3. The effect of MSP supplementation on the growth of calf mucosa and the relative expression of small intestinal enzyme activity mRNA Supplemental Table S4. Pearson correlation coefficients between the growth of calf mucosa and the relative expression of jejunum enzyme activity mRNA [file JSFA-102-4373-s001.docx]

**Effects of multispecies probiotic supplementation on the epithelial growth and development of the intestinal tract in neonatal calves infected by *E. coli* K99**

**Yanyan Wu**^1^**, Cunxi Nie**^1^**, Ruiqing Luo**^2^**, Hongli Chen**^2^**, Junli Niu**^1^**, Chen Chen ^1^, Xue Bai ^1^, Wenju Zhang***^1^

^1^College of Animal Science & Technology, Shihezi University, Shihezi, 832003, China;

^2^Xinjiang Tianshan Junken Animal Husbandry Co., Ltd. Shihezi, 832003, China

*^1^Corresponding author: Wenju Zhang, College of Animal Science & Technology, Shihezi University, North 4th Road, Shihezi, 832003, China

1. mail: [zhangwj1022@sina.com](mailto:zhangwj1022@sina.com))

**Supplementary Materials**: Table S1. Ingredient composition and nutrient levels of starter (DM basis), Table S2. Primer sequences and annealing temperature, Table S3. The effect of MSP supplementation on the growth of calf mucosa and the relative expression of small intestinal enzyme activity mRNA, Table S3. Pearson correlation coefficients between the growth of calf mucosa and the relative expression of jejunum enzyme activity mRNA.

Supplemental Table S1. Ingredient composition and nutrient levels of starter (DM basis)

| Item | Value |
| --- | --- |
| Ingredient, g / kg of DM |  |
| Corn | 55.20 |
| Soybean meal^1^ | 18.50 |
| Corn gluten meal | 10.00 |
| DGGS^2^ | 13.00 |
| Limestone | 1.80 |
| NaCl | 0.50 |
| Premix^3^ | 1.00 |
| Total | 100.00 |
| Chemical analysis |  |
| DM, g / kg | 87.33 |
| CP, g / kg | 19.92 |
| Ether extract, g / kg | 4.64 |
| ADF, g / kg | 6.02 |
| NDF, g / kg | 16.53 |
| Ash, g / kg | 5.38 |
| Calcium | 1.15 |
| Phosphorus | 0.58 |

^1^Soybean meal: 89.1% DM and 42.6% CP

^2^DDGS = distiller’s dried grains with solubles are the nutrient rich co-product of dry-milled ethanol production.

^3^ Premix provides the following per kg of the starter diet: VA 15,000 IU, VD 5000 IU, VE 50 mg, Fe 90 mg, Cu 12.5 mg, Mn 30 mg, Zn 90 mg, Se 0.3 mg, I 1.0 mg, Co 0.5 mg.

Supplemental Table S2. Primer sequences and annealing temperature

| Gene^1^ | Forward primer sequence 5’-3’  Reverse primer sequence 5’-3’ | Tm^2^ (°C) | length (bp) | Ta^3^ (°C) | Mean  quantification cycle (Cq) | Source | GenBank accession no. |
| --- | --- | --- | --- | --- | --- | --- | --- |
| Reference gene | | | | | | | |
| *RPL32* | AGGGTGCGCAGAAGATTCAA | 60 | 188 | 60 | 21.18 | Kochr et al.,2019 | NM_001034783.2 |
|  | TTGGAGGAGACGTTGTGAGC | 60 |  |  |  |  |  |
| Genes of interest | | | | | | | |
| *GHR* | AGT GAA GCC ACA CCA GCT TT | 55 | 154 | 55 | 24.77 | Current paper | NM_176608.1 |
|  | TAG CCC CAT CTG TCC AGT GA | 60 |  |  |  |  |  |
| *IGF1* | TCGCATCTCTTCTATCTGGCCCTGT | 65 | 240 | 60 | 26.21 | Pfaffl et al., 2002 | [NM_001077828.1](http://www.ncbi.nlm.nih.gov/nucleotide/41386724?report=gbwithparts) |
|  | GCAGTACATCTCCAGCCTCCTCAGA | 66 |  |  |  |  |  |
| *IGFBP2* | CACCGGCAGATGGGCAA | 58 | 142 | 58 | 21.92 | Current paper | NM_174555.1 |
|  | GAAGGCGCATGGTGGAGAT | 59 |  |  |  |  |  |
| *IGFBP3* | AAAGGTCATGCCAAGGACAG | 60 | 139 | 56 | 23.26 | Schäff et al., 2018 | NM_174556.1 |
|  | GGTTCAGCGTGTCTTCCATT | 60 |  |  |  |  |  |
| *IGF1R* | TTTCTCAATGAGGCCTCGGT | 60 | 223 | 60 | 24.88 | Current paper | NM_000875 |
|  | CTGCAATCTCACCAGCCATC | 60 |  |  |  |  |  |
| *INSR* | TCCTCAAGGAGCTGGAGGAGT | 54 | 163 | 62 | 24.91 | Current paper | AJ320235.1 |
|  | GCTGCTGTCACATTCCCCA | 68 |  |  |  |  |  |
| SLC5A1 | CAGTCAGCACAGAGTGGACAG | 60 | 308 | 60 | 24.86 | Current paper | NM_174606.2 |
|  | AAGAGGGAGACAGCCAGGAT |  |  |  |  |  |  |
| *PC* | ACACCAACTACCCCGACAATG | 60 | 353 | 60 | 24.43 | Current paper | AY185595 |
|  | CAGCGGGAGGTCAGGGAAG | 69 |  |  |  |  |  |
| *PCK1* | ATGACAACTGCTGGTTGGCT | 53 | 367 | 60 | 26.37 | Current paper | NM_174737.2 |
|  | TGGAGGCACTTGACGAACTC | 60 |  |  |  |  |  |
| *PCK2* | GCCGTAGACCCAAAGGAGTC | 60 | 181 | 60 | 25.42 | Current paper | NM_001205594.1 |
|  | TCAAGGTAGCGCCCAAAGTT | 58 |  |  |  |  |  |
| *PCCA* | AACGTTTGGCAGCAGAAGAT | 56 | 190 | 53 | 23.33 | Graber et al., 2010 | NM_001083509 |
|  | TGACAGGGTAGCCAATTTCC | 58 |  |  |  |  |  |
| *LCT* | AAGGTGGCGTCATCTCCATC | 60 | 217 | 60 | 23.13 | Current paper | NM_001205787.1 |
|  | TCAGGGAGCCGAGACTTGTT | 60 |  |  |  |  |  |
| *MGAM* | TGGCATGCTTGAGTTCAACC | 58 | 240 | 60 | 23.90 | Current paper | XM_015463156 |
|  | GGCAGAAGGGTATAGCGGAT | 60 |  |  |  |  |  |
| *SI* | GGGTGCAGAAACAGTGAGTG | 60 | 194 | 60 | 23.12 | Current paper | NM_001114189 |
|  | GGTTCTTTCGGCTTGCTGTT | 58 |  |  |  |  |  |
| *LDHA* | GAGCCGTCTGAATTTGGTCC | 60 | 207 | 60 | 22.27 | Current paper | NM_174099 |
|  | AGATAACGGAAGCGAGCTGA | 60 |  |  |  |  |  |

^1^Ribosmal protein L32 (***RPL32***); growth hormone receptor (***GHR***); insulin-like growth factor 1 (***IGF1***); insulin-like growth factor binding protein 2 (***IGFBP2***); insulin-like growth factor binding protein 3 (***IGFBP3***); insulin-like growth factor 1 receptor (***IGF1R***); insulin receptor (***INSR***); pyruvate carboxylase (***PC***); cytosolic phosphoenolpyruvate carboxykinase (***PCK1***); mitochondrial phosphoenolpyruvate carboxykinase (***PCK2***); propionyl- CoA carboxylase α (***PCCA***). sodium/glucose cotransporter 1 (***SLC5A1***); lactase (***LCT***); maltase-glucoamylase (***MGAM***); sucrase-isomaltase (***SI***); lactate dehydrogenase A (***LDHA***).

^2^Tm = melting temperature of the primer

^3^Ta = annealing temperature, held for 30 s

Supplemental Table S3. The effect of MSP supplementation on the growth of calf mucosa and the relative expression of small intestinal enzyme activity mRNA

| Items^1,2^ | Treatment | | |  | P-value |  |
| --- | --- | --- | --- | --- | --- | --- |
|  | C | D | MSP | SEM | Gut segmen | Trt |
| *GHR* |  |  |  |  |  |  |
| Duodenum | 1.00 | 1.11 | 0.40 | 0.11 | 0.01 | 0.01 |
| Jejunum | 1.00^b^ | 2.13^b^ | 7.60^a^ | 1.32 |  |  |
| Ileum | 1.00 | 0.59 | 1.72 | 0.21 |  |  |
| *IGF1* |  |  |  |  |  |  |
| Duodenum | 1.00 | 2.19^a^ | 2.42^a^ | 0.19 | <0.01 | <0.01 |
| Jejunum | 1.00^b^ | 0.79^b^ | 4.92^a^ | 0.42 |  |  |
| Ileum | 1.00^b^ | 0.79^b^ | 1.46^a^ | 1.83 |  |  |
| *IGFBP2* |  |  |  |  |  |  |
| Duodenum | 1.00^b^ | 2.72^ab^ | 1.92^a^ | 0.31 | <0.01 | <0.01 |
| Jejunum | 1.00 | 0.81 | 3.27 | 0.30 |  |  |
| Ileum | 1.00^b^ | 1.31^b^ | 11.74^a^ | 1.32 |  |  |
| *IGFBP3* |  |  |  |  |  |  |
| Duodenum | 1.00^b^ | 4.69^a^ | 4.64^a^ | 0.63 | 0.01 | 0.01 |
| Jejunum | 1.00^b^ | 4.97^b^ | 21.86^a^ | 2.93 |  |  |
| Ileum | 1.00^b^ | 0.21^b^ | 5.59^a^ | 0.83 |  |  |
| *IGF1R* |  |  |  |  |  |  |
| Duodenum | 1.00 | 4.29 | 5.76 | 0.95 | 0.10 | 0.10 |
| Jejunum | 1.00^b^ | 0.65^b^ | 9.9^a^ | 1.07 |  |  |
| Ileum | 1.00^b^ | 0.45^b^ | 6.76^a^ | 0.75 |  |  |
| *INSR* |  |  |  |  |  |  |
| Duodenum | 1.00 | 0.99 | 1.69 | 3.5 | 0.02 | 0.07 |
| Jejunum | 1.00 | 14.12 | 12.21 | 0.14 |  |  |
| Ileum | 1.00 | 0.56 | 15.37 | 3.17 |  |  |
| *PC* |  |  |  |  |  |  |
| Duodenum | 1.00 | 1.63^a^ | 1.93^a^ | 0.17 | 0.06 | 0.07 |
| Jejunum | 1.00^b^ | 2.66^a^ | 1.87^ab^ | 0.25 |  |  |
| Ileum | 1.00^ab^ | 1.73^a^ | 0.70^b^ | 0.17 |  |  |
| *PCK1* |  |  |  |  |  |  |
| Duodenum | 1.00 | 0.92 | 1.24 | 0.13 | <0.01 | <0.01 |
| Jejunum | 1.00^b^ | 0.47^b^ | 7.8^a^ | 0.95 |  |  |
| Ileum | 1.00^b^ | 1.36^b^ | 23.76^a^ | 3.89 |  |  |
| *PCK2* |  |  |  |  |  |  |
| Duodenum | 1.00^c^ | 2.24^b^ | 4.47^a^ | 0.36 | <0.01 | <0.01 |
| Jejunum | 1.00^b^ | 2.12^b^ | 20.28^a^ | 2.15 |  |  |
| Ileum | 1.00^b^ | 0.82^b^ | 16.49^a^ | 1.79 |  |  |
| *PCCA* |  |  |  |  |  |  |
| Duodenum | 1.00^b^ | 4.63^a^ | 1.70^b^ | 0.46 | <0.01 | <0.01 |
| Jejunum | 1.00^b^ | 4.01^b^ | 21.55^a^ | 3.65 |  |  |
| Ileum | 1.00 | 0.01 | 0.11 | 0.33 |  |  |
| *SLC5A1* |  |  |  |  |  |  |
| Duodenum | 1.00^b^ | 2.63^a^ | 1.62^ab^ | 0.30 | 0.02 | 0.07 |
| Jejunum | 1.00^b^ | 2.01^b^ | 15.81^a^ | 2.08 |  |  |
| Ileum | 1.00^b^ | 0.75^b^ | 6.33^a^ | 8.77 |  |  |
| *LCT* |  |  |  |  |  |  |
| Duodenum | 1.00 | 0.76 | 0.60 | 0.2 | <0.01 | <0.01 |
| Jejunum | 1.00^b^ | 0.83^b^ | 3.13^a^ | 0.27 |  |  |
| Ileum | 1.00 | 0.32 | 2.55 | 0.24 | <0.01 | <0.01 |
| *MGAM* |  |  |  |  |  |  |
| Duodenum | 1.00^b^ | 3.05^b^ | 11.49^a^ | 1.18 | <0.01 | <0.01 |
| Jejunum | 1.00^b^ | 4.01^b^ | 72.15^a^ | 8.36 |  |  |
| Ileum | 1.00^b^ | 1.55^b^ | 58.67^a^ | 6.88 |  |  |
| *SI* |  |  |  |  |  |  |
| Duodenum | 1.00 | 4.37 | 2.12 | 1.02 | <0.01 | <0.01 |
| Jejunum | 1c | 15.70^b^ | 29.39^a^ | 3.55 |  |  |
| Ileum | 1.00^b^ | 6.74^ab^ | 13.6^a^ | 2.35 |  |  |
| *LDHA* |  |  |  |  |  |  |
| Duodenum | 1.00 | 4.03 | 2.71 | 0.44 | <0.01 | 0.01 |
| Jejunum | 1^ab^ | 0.69^b^ | 1.43^a^ | 0.14 |  |  |
| Ileum | 1.00^b^ | 12.44^b^ | 57.17^a^ | 6.87 |  |  |

^a-c^Different letters within a row indicate significant differences (P ≤ 0.05).

^1^ Values are presented as LSM with SE; n = 6 per group.

^2^ *GHR* = growth hormone receptor; *IGF1* = insulin-like growth factor 1; *IGFBP2* = insulin-like growth factor binding protein 2; *IGFBP3* = insulin-like growth factor binding protein 3; *IGF1R* = insulin-like growth factor 1 receptor; *INSR* = insulin receptor; *PC* = pyruvate carboxylase; *PCK1* = cytosolic phosphoenolpyruvate carboxykinase; *PCK2* = mitochondrial phosphoenolpyruvate carboxykinase; *PCCA* = propionyl-CoA carboxylase; *SLC5A1* = sodium-dependent glucose cotransporter 1; *LCT* = lactase; *MGAM* = maltase-glucoamylase; *SI* = sucrase-isomaltase; *LDHA* = lactate dehydrogenase A; *LDHB* = lactate dehydrogenase B.

Supplemental Table S4. Pearson correlation coefficients between the growth of calf mucosa and the relative expression of jejunum enzyme activity mRNA

| Items1,2 | Villus height | Crypt depth | Villus height/crypt depth | Intestinal wall thickness | Ca2+-Mg2+-AT Pase | Na-K-AT Pase | CK | Maltase | Lactase | Lipase | Try | AMS |
| --- | --- | --- | --- | --- | --- | --- | --- | --- | --- | --- | --- | --- |
| *GHR* |  |  |  |  |  |  |  |  |  |  |  |  |
| Duodenum | -0.07 | -0.45 | 0.24 | 0.68 | 0.07 | -0.26 | 0.11 | -0.11 | 0.08 | 0.09 | 0.35 | 0.08 |
| Jejunum | 0.11 | -0.60 | 0.40 | -0.37 | 0.92* | 0.04 | -0.38 | -0.04 | 0.27 | 0.26 | 0.45 | 0.08 |
| Ileum | -0.17 | 0.29 | -0.18 | -0.969** | 0.86 | 0.47 | 0.77 | 0.07 | 0.53 | 0.62 | -0.10 | 0.10 |
| *IGF1* |  |  |  |  |  |  |  |  |  |  |  |  |
| Duodenum | -0.18 | -0.52 | 0.28 | -0.74 | -0.06 | -0.17 | 0.73 | 0.08 | -0.14 | 0.32 | -0.32 | 0.56 |
| Jejunum | 0.93* | 0.59 | 0.83* | -0.35 | 0.57 | -0.73 | 0.86* | -0.39 | -0.79 | 0.27 | 0.00 | 0.75 |
| Ileum | 0.35 | 0.32 | 0.08 | 0.21 | 0.33 | 0.44 | -0.01 | 0.18 | 0.46 | 0.37 | -0.63 | 0.43 |
| *IGFBP2* |  |  |  |  |  |  |  |  |  |  |  |  |
| Duodenum | 0.05 | -0.20 | 0.14 | 0.91** | 0.06 | -0.24 | -0.23 | -0.15 | 0.09 | 0.01 | 0.37 | -0.10 |
| Jejunum | -0.33 | -0.37 | -0.25 | 0.04 | 0.63 | -0.11 | -0.36 | 0.31 | 0.29 | 0.24 | 0.39 | 0.20 |
| Ileum | 0.34 | 0.21 | 0.11 | 0.28 | 0.51 | 0.20 | 0.03 | 0.58 | 0.27 | 0.31 | -0.65 | 0.08 |
| *IGFBP3* |  |  |  |  |  |  |  |  |  |  |  |  |
| Duodenum | -0.13 | 0.70 | 0.10 | -0.95** | -0.10 | 0.02 | 0.56 | 0.13 | -0.15 | 0.23 | -0.42 | 0.41 |
| Jejunum | -0.06 | -0.15 | -0.01 | -0.47 | 0.49 | -0.29 | -0.18 | -0.70 | -0.19 | 0.93** | -0.56 | -0.47 |
| Ileum | 0.45 | -0.10 | 0.46 | -0.78 | 0.93* | 0.61 | 0.34 | -0.10 | 0.73 | 0.54 | -0.40 | -0.33 |
| *IGF1R* |  |  |  |  |  |  |  |  |  |  |  |  |
| Duodenum | -0.07 | -0.16 | 0.02 | 0.92** | 0.14 | -0.16 | -0.20 | -0.29 | 0.21 | 0.05 | 0.27 | -0.12 |
| Jejunum | 0.65 | 0.37 | 0.60 | -0.39 | 0.55 | -0.54 | 0.52 | 0.06 | -0.11 | -0.09 | 0.73 | 0.91* |
| Ileum | 0.35 | 0.32 | 0.08 | 0.21 | 0.33 | 0.44 | -0.01 | 0.18 | 0.46 | 0.37 | -0.63 | 0.43 |
| *INSR* |  |  |  |  |  |  |  |  |  |  |  |  |
| Duodenum | 0.15 | 0.64 | -0.34 | -0.22 | -0.02 | 0.29 | -0.46 | 0.03 | 0.00 | -0.22 | -0.18 | -0.33 |
| Jejunum | -0.29 | -0.13 | -0.32 | -0.04 | 0.68 | -0.31 | -0.16 | 0.00 | -0.09 | 0.55 | -0.13 | 0.04 |
| Ileum | 0.55 | -0.13 | 0.55 | -0.76 | 0.75 | 0.77 | 0.17 | -0.49 | 0.86* | 0.49 | -0.38 | -0.15 |
| *PC* |  |  |  |  |  |  |  |  |  |  |  |  |
| Duodenum | 0.10 | 0.53 | -0.28 | -0.53 | -0.05 | 0.28 | -0.24 | 0.09 | -0.05 | -0.14 | -0.30 | -0.17 |
| Jejunum | -0.05 | -0.08 | -0.05 | -0.47 | 0.79 | -0.48 | -0.10 | -0.46 | -0.14 | 0.88* | -0.22 | -0.10 |
| Ileum | -0.35 | 0.22 | -0.31 | -0.63 | 0.34 | 0.01 | 0.57 | 0.05 | -0.04 | -0.03 | 0.21 | -0.22 |
| *PCK1* |  |  |  |  |  |  |  |  |  |  |  |  |
| Duodenum | 0.53 | 0.50 | 0.01 | 0.47 | 0.09 | -0.05 | -0.86* | 0.28 | -0.12 | -0.70 | 0.44 | -0.54 |
| Jejunum | -0.05 | -0.08 | -0.05 | -0.47 | 0.79 | -0.48 | -0.10 | -0.46 | -0.14 | 0.88* | -0.22 | -0.10 |
| Ileum | -0.12 | 0.61 | -0.32 | -.880* | 0.62 | 0.73 | 0.82* | 0.01 | 0.75 | 0.73 | -0.34 | 0.52 |
| *PCK2* |  |  |  |  |  |  |  |  |  |  |  |  |
| Duodenum | 0.20 | 0.26 | -0.06 | -0.05 | -0.10 | 0.36 | -0.17 | 0.51 | -0.12 | -0.58 | 0.71 | -0.50 |
| Jejunum | 0.27 | 0.16 | 0.23 | -0.07 | 0.72 | -0.51 | 0.34 | -0.33 | -0.72 | 0.63 | -0.49 | 0.21 |
| Ileum | -0.54 | 0.33 | -0.53 | -0.31 | -0.34 | 0.03 | 0.31 | -0.39 | -0.08 | -0.05 | 0.43 | 0.50 |
| *PCCA* |  |  |  |  |  |  |  |  |  |  |  |  |
| Duodenum | 0.03 | 0.00 | -0.02 | 0.97** | 0.11 | -0.11 | -0.37 | -0.20 | 0.17 | -0.08 | 0.36 | -0.25 |
| Jejunum | 0.47 | 0.08 | 0.51 | -0.21 | 0.63 | -0.36 | 0.29 | 0.24 | -0.03 | -0.11 | 0.73 | 0.83* |
| Ileum | -0.73 | -0.09 | -0.49 | -0.16 | 0.12 | -0.63 | 0.32 | 0.44 | -0.56 | 0.04 | 0.65 | -0.05 |
| *SLC5A1* |  |  |  |  |  |  |  |  |  |  |  |  |
| Duodenum | -0.69 | 0.30 | 0.27 | -0.03 | -0.23 | 0.75 | 0.06 | 0.02 | 0.42 | 0.14 | 0.54 | 0.26 |
| Jejunum | -0.64 | -0.08 | 0.76 | 0.10 | 0.25 | 0.88** | 0.34 | 0.93** | 0.23 | 0.33 | 0.15 | 0.24 |
| Ileum | -0.58 | 0.79 | 0.83* | -0.03 | -0.38 | -0.12 | 0.59 | 0.45 | 0.30 | 0.22 | 0.01 | 0.30 |
| *LCT* |  |  |  |  |  |  |  |  |  |  |  |  |
| Duodenum | -0.30 | -0.22 | -0.04 | 0.03 | 0.14 | -0.26 | 0.22 | -0.58 | 0.21 | 0.60 | -0.74 | 0.46 |
| Jejunum | -0.21 | -0.10 | -0.18 | -0.03 | -0.68 | 0.44 | -0.29 | 0.22 | 0.69 | -0.56 | 0.46 | -0.22 |
| Ileum |  |  |  |  |  |  |  |  |  |  |  |  |
| *MGAM* |  |  |  |  |  |  |  |  |  |  |  |  |
| Duodenum | 0.14 | 0.27 | -0.13 | -0.07 | -0.07 | 0.41 | -0.14 | 0.45 | -0.06 | -0.55 | -0.51 | -0.51 |
| Jejunum | 0.30 | 0.19 | 0.30 | 0.26 | -0.13 | 0.75 | -0.55 | 0.36 | -0.40 | -0.73 | 0.69 | -0.50 |
| Ileum | 0.37 | 0.23 | -0.32 | -0.34 | 0.01 | 0.35 | -0.32 | -0.10 | -0.05 | 0.43 | 0.50 | 0.33 |
| *SI* |  |  |  |  |  |  |  |  |  |  |  |  |
| Duodenum | -0.17 | -0.51 | 0.27 | -0.67 | -0.07 | -0.14 | 0.70 | 0.11 | -0.13 | 0.28 | -0.22 | 0.50 |
| Jejunum | -0.01 | -0.69 | 0.31 | 0.01 | 0.26 | 0.42 | -0.46 | 0.33 | 0.43 | -0.27 | 0.57 | 0.06 |
| Ileum | -0.09 | -0.31 | 0.08 | 0.10 | 0.14 | -0.51 | -0.03 | 0.47 | -0.45 | -0.24 | 0.19 | -0.66 |
| *LDHA* |  |  |  |  |  |  |  |  |  |  |  |  |
| Duodenum | 0.13 | 0.65 | -0.37 | 0.03 | -0.01 | 0.28 | -0.54 | -0.04 | 0.05 | -0.19 | -0.12 | -0.38 |
| Jejunum | -0.32 | -0.39 | -0.20 | -0.46 | 0.54 | -0.14 | -0.48 | -0.40 | 0.23 | 0.78 | -0.16 | -0.43 |
| Ileum | 0.37 | 0.23 | 0.10 | 0.57 | -0.79 | 0.07 | -0.37 | -0.20 | -0.15 | -0.74 | -0.36 | -0.19 |

*P<0.05, **P<0.01.
